# SLC7A5 promotes vascular remodeling in the rat carotid artery following balloon injury through PI3K/Akt signaling pathway

**DOI:** 10.3389/fphar.2026.1857850

**Published:** 2026-07-14

**Authors:** Yu Bai, Sixuan Chen, Yue Wang, Qiming Wu, Dechun Yin

**Affiliations:** 1 Department of Cardiology, The First Affiliated Hospital of Harbin Medical University, Harbin, China; 2 Department of Geriatrics, The First Affiliated Hospital of Harbin Medical University, Harbin, China

**Keywords:** JPH203, PI3K/AKT, SLC7A5, vascular remodeling, VSMCs

## Abstract

**Background:**

Vascular remodeling is a central pathological feature of cardiovascular diseases and is driven in part by vascular smooth muscle cell (VSMC) proliferation, migration, and phenotypic switching. The solute carrier family 7 member 5 (SLC7A5), a key amino acid transporter, has been implicated in cellular growth and metabolic regulation, but its role in vascular remodeling remains unclear. We investigated the contribution of SLC7A5 to VSMC activation and the underlying signaling mechanisms.

**Methods/Results:**

Differential expression analysis of the GSE220512 dataset revealed significant upregulation of Slc7a5 in mouse carotid arteries at Day 7 following wire injury compared with uninjured Day 0 controls (log_2_FC = 2.39, adjusted *P* = 0.0064). In a rat carotid artery balloon injury model, SLC7A5 expression was upregulated and localized to the medial layer. *In vitro*, platelet-derived growth factor-BB (PDGF-BB) increased SLC7A5 expression in VSMCs. Functional studies showed that siRNA-mediated knockdown of SLC7A5 attenuated PDGF-BB–induced VSMC proliferation, migration, and phenotypic switching, as evidenced by reduced PCNA and MMP2 expression, decreased DNA synthesis, impaired migration, and partial restoration of α-smooth muscle actin expression. Mechanistically, SLC7A5 knockdown reduced phosphorylation of phosphatidylinositol 3-kinase (PI3K) and Akt without affecting total protein levels. Pharmacological inhibition of SLC7A5 using JPH203 produced similar effects *in vitro* and reduced neointimal hyperplasia and improved vascular function *in vivo*, with suppression of PI3K/Akt signaling.

**Conclusion:**

These findings identify SLC7A5 as a critical regulator of VSMC activation and vascular remodeling. SLC7A5 promotes proliferative and migratory responses, at least in part through activation of the PI3K/Akt signaling pathway. Targeting SLC7A5 may represent a potential therapeutic strategy for vascular remodeling–associated cardiovascular diseases.

## 1 Introduction

Cardiovascular diseases are the leading cause of global morbidity and mortality (Zhou et al., 2020; Nathon and Naveed, 2023). Vascular remodeling represents a shared pathological basis of hypertension, atherosclerosis, and post-interventional restenosis, characterized by maladaptive structural reorganization of vascular endothelial and smooth muscle cells in response to hemodynamic stress or injury (Qi et al., 2019; Zhang Y. et al., 2024; Du et al., 2025). Current clinical interventions, including drug-eluting stents and antihypertensive drugs, relieve symptoms but fail to fully block or reverse vascular remodeling, as reflected by persistent in-stent restenosis and progressive atherosclerosis (Lahmann and Joner, 2020). This unmet clinical need highlights the critical gaps in our mechanistic understanding and the urgent demand for novel therapeutic targets.

Emerging evidence reveals that vascular remodeling is not only driven by abnormal cell proliferation and migration but also controlled by metabolic reprogramming (Wenjuan et al., 2020; Marissa et al., 2023; RuLi et al., 2024). Similar to the Warburg effect in cancer, proliferating VSMCs exhibit increased nutrient dependency, especially for amino acids (Martina et al., 2005; Tong et al., 2023). As a key transporter for large neutral amino acids and the primary mediator of leucine uptake, SLC7A5 serves as both a nutrient gatekeeper and a critical sensor linking extracellular metabolic signals to intracellular signaling networks (Yoshikatsu, 2021; Yang et al., 2026).

In cancer, SLC7A5 promotes cell proliferation via regulating mTOR signaling (Zhou et al., 2024), yet its role in cardiovascular diseases, particularly vascular remodeling, remains largely unknown. The PI3K/Akt pathway is a well-established core regulator of cell survival, proliferation, and migration, and its hyperactivation is a major driver of pathological vascular remodeling (Dang et al., 2021; Jianqiu et al., 2022). While the importance of PI3K/Akt signaling in vascular pathology is well established, the upstream modulators that link metabolic cues to this pathway are not fully defined.

Given that amino acid availability is a key determinant of cellular signaling and metabolic activity (Dirk et al., 2018; Bolin et al., 2024), it is plausible that SLC7A5 may influence vascular remodeling by modulating PI3K/Akt pathway activation. However, whether SLC7A5 exerts a protective or detrimental effect in this context, and the precise mechanisms involved, remain unclear. Therefore, in the present study, we employed a rat carotid artery balloon injury model to investigate the role of SLC7A5 in vascular remodeling. We further explored whether SLC7A5 modulates neointimal formation through the PI3K/Akt signaling pathway. Elucidating these mechanisms may provide new insights into the metabolic regulation of vascular remodeling and identify potential therapeutic targets for preventing restenosis.

## 2 Materials and methods

### 2.1 Bioinformatic analysis of public RNA-Seq data

For RNA-seq or MACE-seq count data, differential expression analysis was performed using the DESeq2 R package, while for microarray expression matrices, the limma package was applied. In both analyses, a design matrix was constructed based on sample grouping, and expression data were normalized prior to statistical testing. Low-expression genes were filtered prior to analysis. For RNA-seq/MACE-seq data, size factor normalization was performed using DESeq2, while microarray data were background-corrected and normalized using quantile normalization. *P* values were adjusted for multiple testing using the Benjamini–Hochberg method to control the false discovery rate, yielding adjusted *P* values. Genes with |log2 fold change| > 1 and adjusted *P* value < 0.05 were considered significantly differentially expressed. For candidate gene SLC7A5/Slc7a5, both raw and adjusted *P* values were reported to ensure transparency in statistical interpretation. All analyses were performed in R.

### 2.2 Materials

JPH203 (KYT-0353) provided by Targetmol was dissolved in DMSO to prepare stock solution,and PDGF-BB from MCE (HY-P7278) was reconstituted in sterile normal saline according to the manufacturer’s instructions.

### 2.3 Animal model of carotid artery balloon injury

All animal procedures in this study were conducted in strict accordance with the Health Guide for the Care and Use of Laboratory Animals and were approved by the Animal Care Committee at Harbin Medical University to ensure humane treatment (Ethical approval number: 2025045). For the balloon catheterization of the carotid artery, rats were anesthetized with inhaled isoflurane (induction with 5% and maintenance of anesthesia with 1%–2%) and were injected with buprenorphine (Veterge sic® 0.1 mg/kg, subcutaneous). A standardized carotid artery balloon angioplasty procedure was used to induce vascular injury to the left common carotid artery (LCA), as previously described (Qingwei et al., 2021; Matsushita et al., 2024). The LCA and its internal/external bifurcation were exposed through a midline neck incision, and internal carotid artery was temporarily clamped to prevent backflow. A small arteriotomy was performed in the external carotid branch and a Fogarty 2F balloon catheter (Edwards Lifesciences, Irvine, CA) was inserted into the common carotid, and down to the aortic arch. The balloon was inflated with 0.1–0.2 mL saline and was pulled back through the common carotid with slight rotation to denudate the endothelium. This procedure was repeated 5 times, after which the catheter was deflated and removed, the external carotid artery was ligated permanently, and the skin incision was sutured. Throughout the whole protocol animal’s weights as well as water and food consumption were monitored. Two weeks after the surgery, the rats were anesthetized with continuous vaporized 5% isoflurane, injected with a lethal dose of a mixture of ketamine and xylazine (120 mg/kg and 10 mg/kg, intraperitoneal) and sacrificed by taking a lethal volume of blood from the right ventricle.

### 2.4 Drug administration and experimental grouping

Rats were randomly assigned to three groups: Sham group, Model group, and treatment group, with 6–7 animals in each group. Sample size was estimated based on previous literature, preliminary experimental data, and expected effect sizes in vascular remodeling outcomes, while also considering the principle of minimizing animal use in accordance with ARRIVE 2.0 recommendations. In the Sham group, animals underwent the same surgical procedures without balloon injury. In the Model group, rats were subjected to carotid artery balloon injury without further intervention. In the treatment group, rats received the selective SLC7A5 inhibitor JPH203 following balloon injury. JPH203 was administered at a dose of 25 mg/kg via intraperitoneal injection once daily consecutive 14 days starting from after surgery or 24 h post-injury. The dosage and administration schedule were determined based on previous studies and preliminary experiments (Zheng et al., 2024).

Animals were monitored daily for general health conditions. At the end of the experimental period, all rats were euthanized (Chen et al., 2026), and carotid artery tissues were collected for subsequent analyses.

### 2.5 Vascular ultrasound assessment

Using the Vinno D650 LAB ultrasound imaging equipment, the Carotid artery diameter, peak systolic velocity and end diastolic velocity were measured at 0 and 14 days. Rats were put under anesthesia and positioned supinely on the operating table. After shaving the neck area, applying a gel, and fixing the acquisition probe, the neck vascular image was captured (Zhang J. et al., 2024).

### 2.6 Tissue collection and histological analysis

At the end of the experimental period, rats were euthanized under anesthesia, and carotid artery tissues were carefully harvested. The vessels were rinsed with cold phosphate-buffered saline (PBS) to remove blood and immediately fixed in 4% paraformaldehyde for 24 h. Subsequently, the tissues were dehydrated, embedded in paraffin, and sectioned into 4–5 μm thick slices for further histological analysis (Nicole et al., 2023).

### 2.7 H&E staining

Paraffin-embedded sections were deparaffinized in xylene and rehydrated through a graded ethanol series. The sections were then stained with hematoxylin for nuclear visualization, followed by eosin staining for cytoplasmic components. After dehydration and mounting, the sections were observed under a light microscope. Morphological changes of the carotid artery, including neointimal formation and medial thickness, were evaluated (Gao et al., 2020). The intima-to-media ratio was calculated using ImageJ software.

### 2.8 Immunohistochemistry

For immunohistochemical staining, paraffin-embedded sections were first deparaffinized and rehydrated following the previously described protocol. Antigen retrieval was then carried out by heating the sections in citrate buffer (pH 6.0). Endogenous peroxidase activity was blocked with 3% hydrogen peroxide, followed by blocking with normal serum to reduce nonspecific binding.

The sections were then incubated overnight at 4 °C with primary antibodies against PCNA and MMP2. After washing, the sections were incubated with the corresponding HRP-linked secondary antibodies at room temperature. The antigen–antibody reaction was detected using a DAB substrate kit, followed by hematoxylin counterstaining to visualize nuclei. Finally, the sections were dehydrated, mounted, and imaged under a light microscope.

### 2.9 Measurement of leucine content in vascular media

Vascular tissue samples were collected in sterile 15 mL centrifuge tubes, with a minimum tissue weight of 50 mg per sample. Tissues were homogenized in phosphate-buffered saline (PBS, 0.01 M, pH 7.4) at a ratio of 1 mg tissue per 10 μL PBS. The homogenates were centrifuged at 3,000 rpm for 5 min at 4 °C, and the supernatants were collected for analysis. Leucine levels in vascular media were determined using a commercially available enzyme-linked immunosorbent assay (ELISA) kit (Shanghai Kexin Biotechnology Co., Ltd., Cat. No. CB14385-Ra), according to the manufacturer’s instructions. Briefly, standards or tissue supernatants were added to microplate wells pre-coated with a capture antibody specific for leucine. Leucine present in the samples bound to the immobilized antibody. After incubation, a biotinylated detection antibody was added, followed by washing steps to remove unbound components. Horseradish peroxidase (HRP)-conjugated streptavidin was then added to form the antibody-antigen-enzyme complex. After thorough washing, tetramethylbenzidine (TMB) substrate solution was added for color development. In the presence of HRP, TMB was oxidized to a blue-colored product, which was subsequently converted into a yellow end-product upon acidification. The intensity of the color was proportional to the leucine concentration in the samples. Optical density (OD) was measured at 450 nm using a microplate reader, and leucine concentrations were calculated based on the standard curve. All samples were assayed in triplicate wells, and each experimental group included five animals.

### 2.10 VSMC culture and treatment

Primary VSMCs were isolated from the thoracic aortas of SD rats as previously described with minor modifications (Estar et al., 2025). Briefly, rats were anesthetized and sacrificed according to institutional guidelines. The thoracic aorta was carefully excised under sterile conditions and placed in cold PBS. After removal of surrounding adipose tissue and connective tissue, the aorta was longitudinally opened, and the endothelial layer and adventitia were gently scraped off. The remaining medial layer was cut into small explants (∼1 mm^3^) and placed onto culture dishes. After allowing the tissue to adhere, Dulbecco’s Modified Eagle Medium (DMEM) supplemented with 10% fetal bovine serum and 1% penicillin–streptomycin was added. Explants were maintained at 37 °C in a humidified atmosphere with 5% CO_2_. VSMCs migrated out from the explants within 7–10 days and were subcultured upon reaching 70%–80% confluence. Cells at passages 3–6 were used for subsequent experiments.

### 2.11 siRNA transfection

A gene-silencing small interfering RNA (siRNA) sequence targeting SLC7A5 (si-SLC7A5) and a control siRNA sequence (si-NC) were engineered by GenePharma (China). At a cellular density of 5 × 10^5^ cells/mL, VSMCs were plated into sixwell plates and transfected with 4 μL Lipofectamine 8000 (C0533; Beyotime Biotechnology, China) and 5 μL siRNA for 6 h. After this procedure, the growth medium was replaced with DMEM supplemented with 2% FBS without antibiotics for 18 h before. The efficiency of gene suppression by siRNA intervention was established through Western blotting assays. The strands were subsequently sequenced as follows: si-SLC7A5, Forward:5′- GGA​UCU​UCU​UCU​ACA​UCU​UTT -3′,Reverse:5′- AAG​AUG​UAG​AAG​AAG​AUC​C TT -3′, and si-NC,Forward:5′- UUC​UCC​GAA​CGU​GUC​ACG​UTT -3′Reverse:5′- ACG​UGA​CAC​GUU​CGG​AGA​ATT -3′(Fedoroff et al., 2025).

### 2.12 Cell proliferation and migration assays (EdU and wound healing)

The cell-light EdU Apollo488 *in vitro* kit (Beyotime, C0071s) was used to detect newly synthesized DNA in VSMCs. All procedures are carried out following the manufacturer’s instructions. In short, cells were incubated in EdU contained medium (10 μmol/L) for 2 h, fixed with 4% paraformaldehyde (Biosharp, Hefei, China), and then penetrated by 0.5% Triton X-100 (Biosharp). Cells were incubated with a dye reaction mixture, and the nucleus was stained with DAPI solution. Images were captured using a fluorescence microscope (Zeiss, Jena, Germany) and the fluorescence intensity was calculated with ImageJ.

Cell migration was assessed using a wound healing assay. VSMCs (4 × 105) with different treatments were plated in 6-well plates and grown in DMEM with 10% FBS. Once the cells reached 90%–100% confluence, a scratch was made using a 200 μL pipette tip, and removed the detached cells by rinsing with PBS. The cells were then cultured in serum-free DMEM, and the wound area was observed at 0 and 24 h. The wound healing rate was determined through the following formula:Wound healing rate = [(wound area at 0 h) − (wound area at 24 h)]/(wound area at 0 h) × 100 %.

### 2.13 Immunofluorescence staining

Cells were fixed with 4% paraformaldehyde for 20 min at room temperature, then washed three times with PBS. Permeabilization was performed with 0.25% Triton X-100 in PBS for 10 min. After blocking with 1% BSA, cells were incubated with the primary antibody (1:200) in PBS overnight at 4 °C. Following three washes with PBS, the secondary antibody was applied to the cells for 1 h in the dark. After additional PBS washes, cells were stained with 0.1% DAPI for 5 min at 20 °C. Finally, the cells were analyzed using a fluorescent microscope (Zhang M. et al., 2024).

### 2.14 Western blot analysis

Proteins from vascular tissues and VSMCs were extracted and quantified by BCA assay, separated by SDS-PAGE, and transferred to PVDF membranes. After incubation with primary and secondary antibodies, signals were detected using ECL and analyzed with Image Lab and ImageJ. The primary antibodies included MMP2(1:1,000, ab92536, Abcam), PCNA(1:1,000, ab92552, Abcam), α-SMA (1:1,000, ab7817, Abcam), SLC7A5 (1:1,000, sc-374232, Santa), Phosphorylated PI3K was detected using an antibody that recognizes PI3K p85 (Tyr458) and p55 (Tyr199) phosphorylation (1:1,000, 4228T, CST), PI3K (1:1,000, 4255T, CST), Phospho-Akt (Ser473) (1:1,000, 4060T, CST), AKT (1:1,000, 4691T, CST), β-Tubulin (1:1,000, 10094-1-AP, Proteintech).

Following washing, the bands were incubated with an HRP-conjugated secondary antibody at room temperature for 1 h and analyzed using an electrochemiluminescence (ECL) system (Xinyue et al., 2024).

### 2.15 Statistical analysis

All data were presented as mean ± SEM. Statistical analyses were performed using GraphPad Prism. Comparisons between two groups were conducted using an unpaired Student’s t-test, while comparisons among multiple groups were analyzed by one-way analysis of variance (ANOVA) followed by appropriate *post hoc* tests. A value of *P* < 0.05 was considered statistically significant. The exact *P* values and statistical significance levels were indicated in the corresponding figures. All experiments were performed at least three times independently.

## 3 Results

### 3.1 SLC7A5 is upregulated in vascular remodeling

**FIGURE 1:**
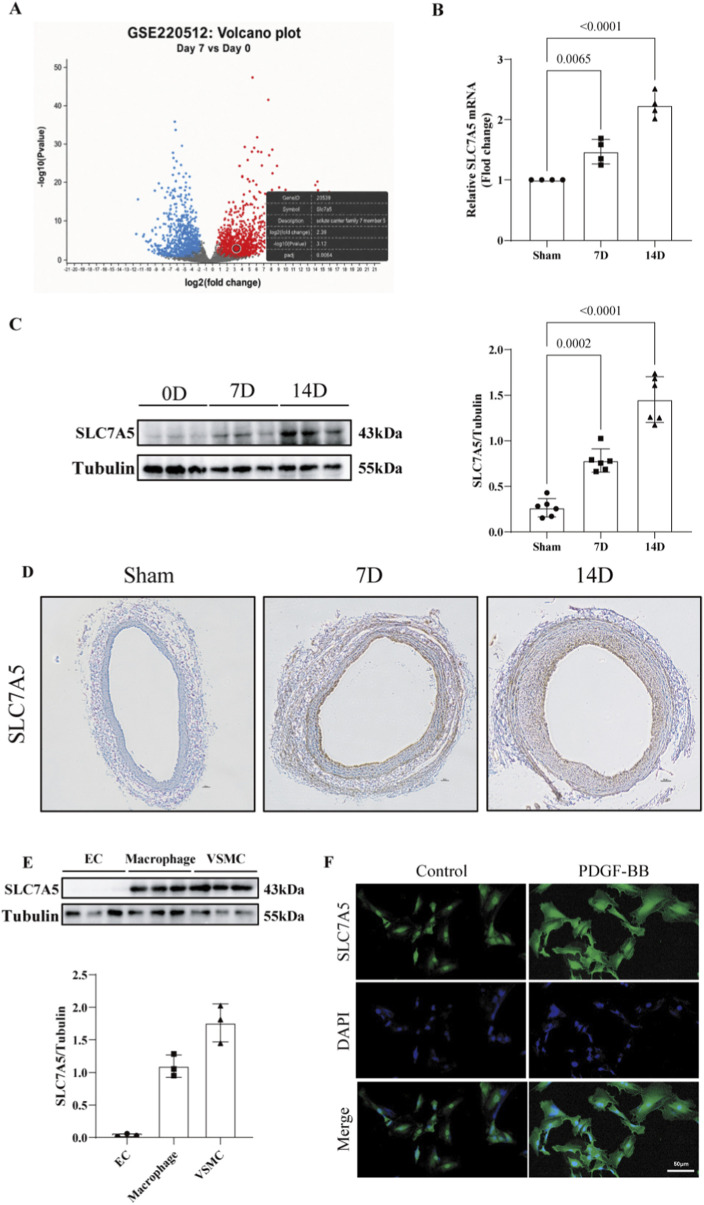
SLC7A5 is upregulated during vascular remodeling. **(A)** Volcano plot of the GSE220512 dataset showing that Slc7a5 is significantly upregulated in neointimal tissue from mouse carotid arteries at Day 7 after wire injury compared with Day 0 controls. **(B)** Quantitative PCR analysis of SLC7A5 mRNA expression at indicated time points. **(C)** Representative Western blot and quantitative analysis showing SLC7A5 protein expression at 0, 7, and 14 days after vascular injury (n = 6). **(D)** Immunohistochemical staining of SLC7A5 in vascular tissues from sham, 7-day, and 14-day groups, showing increased expression in the medial layer during remodeling. bar = 50 μm. **(E)** Western blot and quantification of SLC7A5 expression in endothelial cells (ECs), macrophages, and vascular smooth muscle cells (VSMCs) (n = 3). **(F)** Immunofluorescence staining showing increased SLC7A5 expression in VSMCs following PDGF-BB stimulation compared with control. bar = 50 μm. Data are presented as mean ± SEM. Statistical significance is indicated as shown.

To investigate the regulatory role of SLC7A5 in vascular remodeling, we examined the expression of SLC7A5 in neointimal tissues obtained by laser microdissection from mouse carotid arteries at 0 and 7 days after wire injury (GSE220512). As shown in Figure 1A, compared with the Day0 group, the expression of SLC7A5 in the neointima of carotid arteries was significantly increased in the Day7 group. Therefore, we speculate that SLC7A5 has a regulatory role in vascular remodeling. This observation was validated *in vivo* by detecting the expression of SLC7A5 during vascular remodeling. Quantitative PCR analysis showed that, compared with the sham-operated group, SLC7A5 mRNA levels gradually increased at 7 and 14 days (Figure 1B). Consistently, Western blot analysis showed that SLC7A5 was significantly upregulated over time, suggesting activation of SLC7A5 during vascular remodeling. Histological analysis further supported these findings (Figure 1C). Immunohistochemical staining further confirmed the progressive upregulation of SLC7A5 in injured carotid arteries. As shown in Figure 1D, SLC7A5-positive staining was weak in the sham group but markedly increased at 7 days after wire injury and became more pronounced at 14 days, predominantly within the thickened neointimal region. These findings further support the activation of SLC7A5 during the progression of vascular remodeling.

Vascular remodeling is a complex and dynamic pathological process involving structural and functional alterations of the vascular wall in response to diverse pathological stimuli, including inflammation, oxidative stress, hypertension, and vascular injury. Endothelial dysfunction and inflammatory cell infiltration contribute to the initiation and progression of this process. However, vascular smooth muscle cells (VSMCs) are considered central mediators of vascular remodeling. Under pathological conditions, VSMCs undergo phenotypic switching from a quiescent contractile phenotype to a synthetic phenotype, characterized by enhanced proliferation, migration, and secretory activity. In parallel, VSMCs actively participate in extracellular matrix (ECM) remodeling through altered synthesis and degradation of matrix components, thereby promoting vessel wall thickening, fibrosis, increased vascular stiffness, and disruption of vascular homeostasis, ultimately contributing to the development and progression of cardiovascular diseases (Zenglei et al., 2023; RuLi et al., 2024; Yu et al., 2024; Du et al., 2025). To further explore the cellular basis of SLC7A5 involvement in this process, its expression was assessed across major vascular cell types, including endothelial cells (ECs), VSMCs, and macrophages (Figure 1E). The results demonstrated that SLC7A5 was predominantly expressed in VSMCs, with comparatively lower expression in ECs and macrophages. Given that platelet-derived growth factor-BB (PDGF-BB) is a well-established mediator that promotes VSMC activation and phenotypic switching during vascular remodeling (Xian et al., 2025), *in vitro* experiments were conducted using PDGF-BB stimulation to mimic the remodeling microenvironment. Immunofluorescence staining showed that PDGF-BB treatment markedly increased SLC7A5 expression compared with control conditions (Figure 1F).

Collectively, these findings demonstrate that SLC7A5 is consistently upregulated during vascular remodeling across bioinformatic analysis, *in vivo* models, and *in vitro* experiments, suggesting a potential role in disease progression and providing a foundation for subsequent mechanistic studies.

### 3.2 Knockdown of SLC7A5 inhibited PDGF-BB–induced VSMC proliferation, migration, and phenotypic switching

**FIGURE 2:**
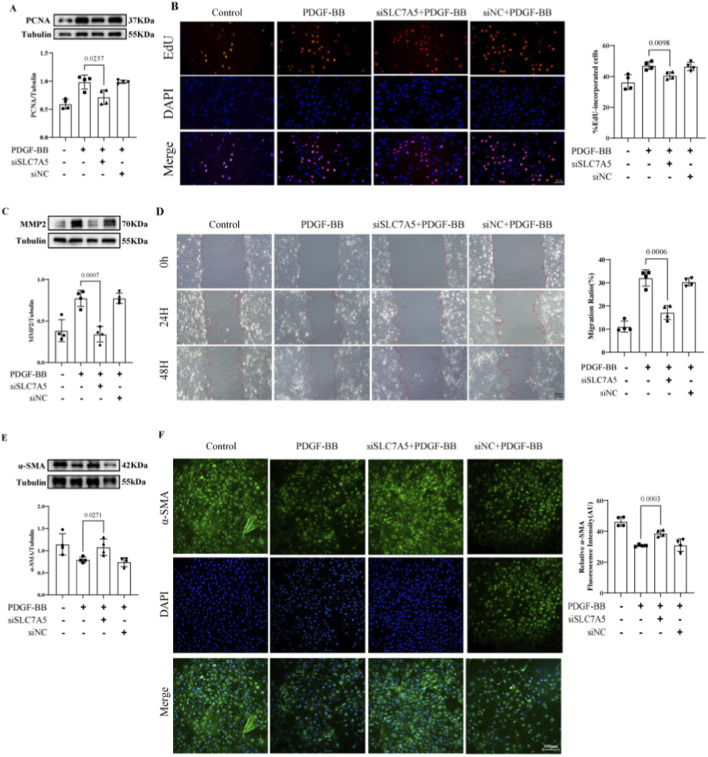
Knockdown of SLC7A5 inhibits PDGF-BB–induced VSMC proliferation, migration, and phenotypic switching. **(A)** Western blot analysis and quantification of PCNA expression in VSMCs under different treatments (n = 4). **(B)** Representative images of EdU staining and quantification of EdU-positive cells (n = 4) bar = 50 μm. **(C)** Western blot analysis and quantification of MMP2 expression (n = 4). **(D)** Representative images of wound healing assays at indicated time points and quantitative analysis of migration rate (n = 4) bar = 50 μm. **(E)** Western blot analysis and quantification of α-SMA expression (n = 4). **(F)** Representative immunofluorescence staining of α-SMA (green) and DAPI (blue), along with quantitative analysis of fluorescence intensity (n = 4) bar = 100 μm. PDGF-BB was used to induce VSMC activation. siSLC7A5 was used to silence SLC7A5 expression, and siNC served as a negative control. Data were presented as mean ± SEM from at four independent experiments. Statistical significance was determined using one-way ANOVA followed by appropriate *post hoc* tests.

The functional role of SLC7A5 in vascular smooth muscle cell (VSMC) activation was examined using siRNA-mediated knockdown under PDGF-BB stimulation. The knockdown efficiency is presented in Supplementary Figure S1. As shown in Figure 2A, Western blot analysis demonstrated that PDGF-BB treatment significantly increased PCNA expression, indicating enhanced proliferative capacity of VSMCs. This effect was markedly attenuated by SLC7A5 knockdown, whereas transfection with negative control siRNA (siNC) had no significant effect. Consistently, EdU incorporation assays further supported these findings (Figure 2B). PDGF-BB stimulation significantly increased the proportion of EdU-positive cells, while silencing of SLC7A5 reduced PDGF-BB-induced DNA synthesis, indicating suppressed cell proliferation. The effect of SLC7A5 on VSMC migration was subsequently evaluated. Western blot analysis showed that PDGF-BB markedly upregulated MMP2 expression, which was significantly reduced following SLC7A5 knockdown (Figure 2C). In parallel, wound healing assays demonstrated that PDGF-BB significantly promoted VSMC migratory capacity, whereas SLC7A5 silencing substantially inhibited this effect (Figure 2D). The role of SLC7A5 in VSMC phenotypic modulation was further assessed by examining the contractile marker α-SMA. Western blot analysis revealed that PDGF-BB treatment decreased α-SMA expression, indicative of a phenotypic switch toward a synthetic state, whereas SLC7A5 knockdown partially restored α-SMA levels (Figure 2E). These findings were corroborated by immunofluorescence staining, which showed a consistent trend in α-SMA expression across different treatment groups (Figure 2F).

Collectively, these results demonstrate that SLC7A5 plays a critical role in mediating PDGF-BB-induced VSMC proliferation, migration, and phenotypic switching. Silencing of SLC7A5 effectively attenuates these pathological processes, suggesting that SLC7A5 contributes to VSMC activation during vascular remodeling.

### 3.3 SLC7A5 knockdown attenuates the activation of the PI3K/Akt signaling pathway induced by PDGF-BB

The PI3K/Akt signaling pathway is widely recognized as a central regulator of VSMCs phenotypic switching, proliferation, and migration in response to vascular injury and growth factor stimulation. Previous studies have demonstrated that activation of PI3K/Akt signaling is a key downstream event of PDGF-BB, driving the transition of VSMCs from a contractile to a synthetic phenotype.

**FIGURE 3:**
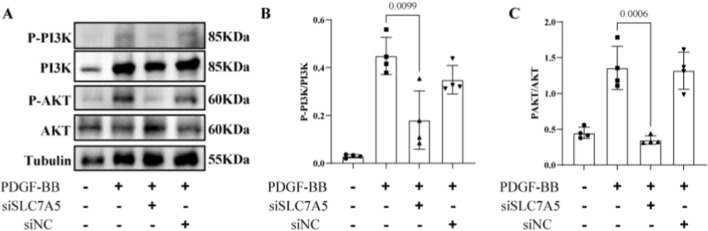
SLC7A5 knockdown attenuates the activation of the PI3K/Akt signaling pathway induced by PDGF-BB. **(A–C)** Western blot analysis of PI3K/Akt signaling pathway–related proteins, including phosphorylated PI3K (p-PI3K), total PI3K, phosphorylated Akt (p-Akt), and total Akt.

To determine whether SLC7A5 is involved in the regulation of this pathway, the activation status of PI3K/Akt signaling was evaluated following SLC7A5 knockdown in PDGF-BB–stimulated VSMCs. As shown in Figure 3A, Western blot analysis showed that PDGF-BB treatment markedly increased PI3K and Akt phosphorylation compared with the control group, indicating robust pathway activation. Notably, siRNA-mediated knockdown of SLC7A5 significantly reduced PDGF-BB–induced phosphorylation of PI3K at the p85 (Tyr458) site and Akt at Ser473, whereas phosphorylation of PI3K at the p55 (Tyr199) site showed no significant change. Total PI3K and Akt protein levels remained unchanged. Quantitative analysis further confirmed that the ratios of p-PI3K (Tyr458)/PI3K and p-Akt (Ser473)/Akt were significantly decreased upon SLC7A5 silencing (Figures 3B,C).

These findings suggest that SLC7A5 plays a critical role in modulating the activation of the PI3K/Akt signaling pathway rather than affecting its basal expression. Given the well-established role of PI3K/Akt signaling in promoting VSMC proliferation, migration, and phenotypic switching, the inhibitory effects observed upon SLC7A5 knockdown indicate that SLC7A5 may function upstream of this pathway to facilitate VSMC activation. Collectively, these results demonstrate that SLC7A5 knockdown attenuates PDGF-BB–induced activation of the PI3K/Akt signaling pathway, providing mechanistic insight into how SLC7A5 regulates VSMC phenotypic modulation.

### 3.4 Pharmacological inhibition of SLC7A5 suppresses PDGF-BB–induced VSMC proliferation, migration, phenotypic switching, and PI3K/Akt signaling

The effect of pharmacological inhibition of SLC7A5 on VSMC activation was further evaluated using JPH203 under PDGF-BB stimulation. CCK-8 assays assessing cell viability across different concentrations of JPH203 are presented in Supplementary Figure S2A, and the corresponding dose–response curve with calculated IC_50_ is shown in Supplementary Figure S2B. To functionally validate the inhibitory effect of JPH203 on SLC7A5 activity in VSMCs, intracellular leucine content was measured using ELISA, since leucine serves as a key substrate of SLC7A5-mediated amino acid transport. As shown in Supplementary Figure S3, PDGF-BB treatment significantly elevated intracellular leucine levels, whereas JPH203 significantly reduced leucine accumulation induced by PDGF-BB, confirming effective inhibition of SLC7A5 transport activity.

**FIGURE 4:**
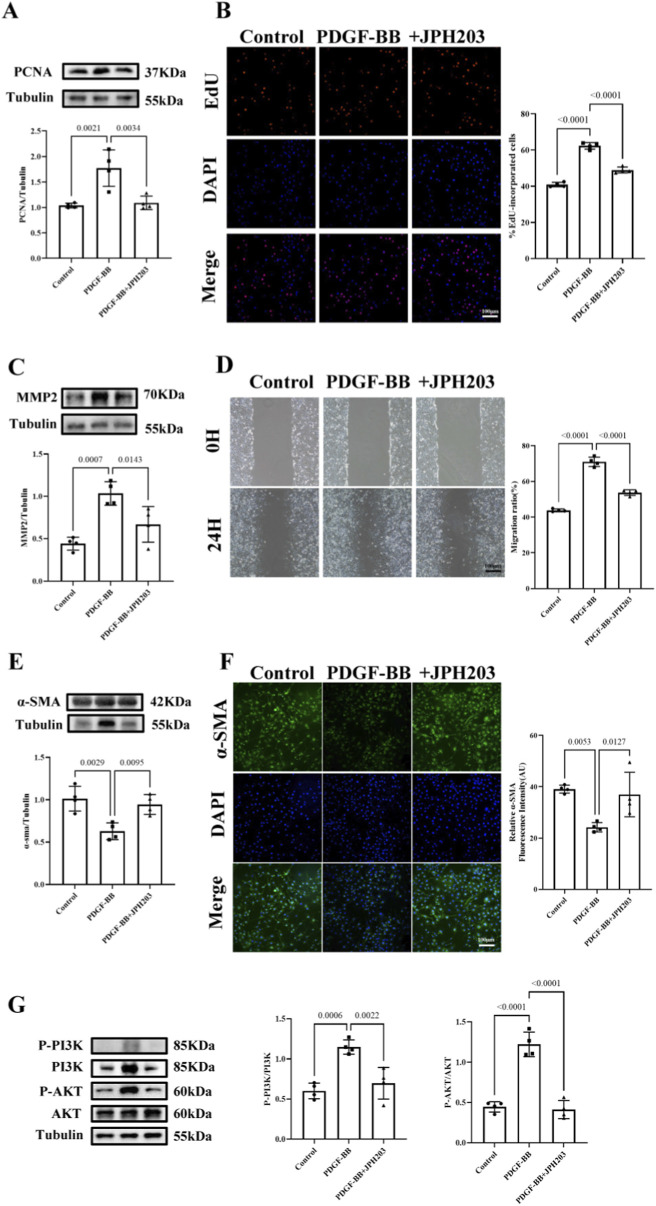
Inhibition of SLC7A5 suppresses PDGF-BB–induced VSMC activation and PI3K/Akt signaling *in vitro*. **(A)** Western blot analysis and quantification of PCNA expression (n = 4). **(B)** Representative images of EdU staining and quantification of proliferating cells. bar = 100 μm. **(C)** Western blot analysis and quantification of MMP2 expression (n=4). **(D)** Representative images of wound healing assays at 0 h and 24 h, and quantitative analysis of migration rate. bar=100 μm. **(E)** Western blot analysis and quantification of α-SMA expression (n=4). **(F)** Representative immunofluorescence staining of α-SMA (green) and DAPI (blue). bar=100 μm. **(G)** Western blot analysis of PI3K/Akt signaling pathway–related proteins, including phosphorylated PI3K (p-PI3K), total PI3K, phosphorylated Akt (p-Akt), and total Akt (n=4). Data are presented as mean ± SEM from at four independent experiments. Statistical significance was determined by one-way ANOVAfollowed by appropriate post hoc tests. bar=100 μm.

As shown in Figure 4A, PCNA expression was markedly elevated following PDGF-BB stimulation and was significantly reduced upon JPH203 treatment, indicating inhibition of VSMC proliferation. EdU incorporation assays further confirmed that JPH203 significantly attenuated PDGF-BB-induced DNA synthesis (Figure 4B). Expression of matrix metalloproteinase-2 (MMP2), a critical regulator of VSMC migration, was significantly elevated following PDGF-BB stimulation and markedly reduced upon JPH203 treatment (Figure 4C). Correspondingly, wound-healing assays revealed that PDGF-BB markedly enhanced VSMC migratory capacity, while JPH203 treatment significantly suppressed cell migration, as indicated by reduced wound closure (Figure 4D).

Alterations in VSMC phenotypic markers were subsequently examined. PDGF-BB treatment significantly reduced the expression of the contractile marker α-SMA, whereas JPH203 partially restored its expression. Both Western blot and immunofluorescence staining yielded consistent results, suggesting that SLC7A5 inhibition attenuated the phenotypic transition of VSMCs toward a synthetic state (Figures 4E,F).

Since PI3K/AKT signaling is critically involved in regulating VSMC biological functions, pathway activation was further assessed. Western blot analysis demonstrated that PDGF-BB significantly increased PI3K and AKT phosphorylation, whereas JPH203 markedly attenuated their activation without affecting total PI3K or AKT expression levels (Figure 4G).

Collectively, these findings indicate that pharmacological inhibition of SLC7A5 by JPH203 suppresses PDGF-BB-induced VSMC proliferation, migration, and phenotypic switching, potentially through inhibition of PI3K/AKT signaling.

### 3.5 Pharmacological inhibition of SLC7A5 alleviates vascular remodeling and suppresses PI3K/Akt signaling *in vivo*

To further evaluate the role of SLC7A5 in vascular remodeling *in vivo*, a rat carotid artery balloon injury model was established, followed by pharmacological inhibition using JPH203.

**FIGURE 5:**
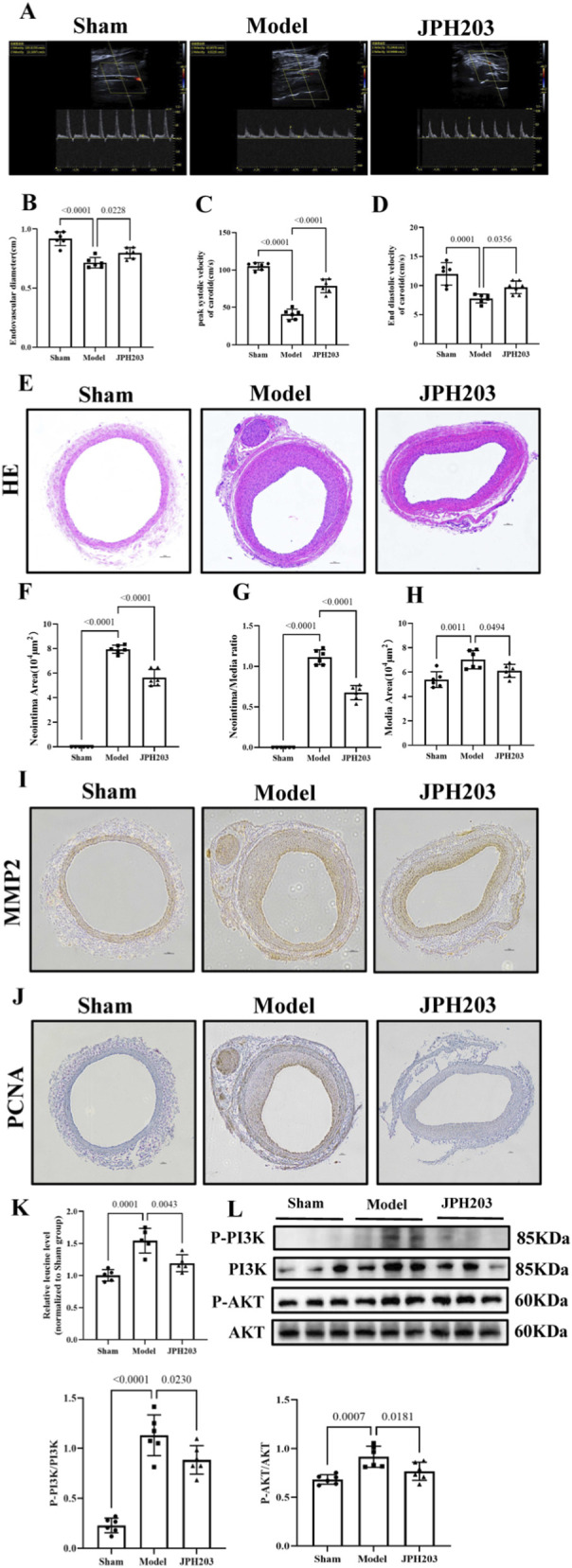
Pharmacological inhibition of SLC7A5 attenuates vascular remodeling and PI3K/Akt signaling *in vivo*. **(A)** Representative vascular ultrasound images of carotid arteries in Sham, Model, and JPH203-treated groups. **(B)** Quantitative analysis of endovascular diameter (n = 6). **(C)** Quantitative analysis of peak systolic velocity (n = 6). **(D)** Quantitative analysis of end-diastolic velocity (n = 6–7). **(E)** Representative H&E staining of carotid artery sections. bar = 50 μm. **(F)** Quantification of neointimal area (n = 6). **(G)** Quantification of intima-to-media ratio (n = 6). **(H)** Quantification of media area (n = 6). **(I)** Representative immunohistochemical staining of MMP2. bar = 50 μm. **(J)** Representative immunohistochemical staining of PCNA. bar = 50 μm. **(K)** Relative leucine level in vascular media normalized to the Sham group (n = 5). **(L)** Western blot analysis of p-PI3K, PI3K, p-Akt, and Akt expression, along with quantitative analysis of p-PI3K/PI3K and p-Akt/Akt (n = 6). Data are presented as mean ± SEM. Statistical significance was determined by one-way ANOVA followed by *post hoc* analysis.

Vascular ultrasound analysis showed that balloon injury significantly impaired vascular function, as evidenced by a reduction in endovascular diameter and peak systolic velocity, along with altered end-diastolic velocity compared with the sham group. Notably, treatment with JPH203 markedly improved these hemodynamic parameters (Figures 5A–D), indicating a protective effect on vascular function. Histological examination by H&E staining revealed pronounced neointimal hyperplasia in the model group, whereas JPH203 treatment significantly reduced neointimal formation (Figure 5E). Quantitative analysis further confirmed that both neointimal area and intima-to-media ratio were significantly decreased in the JPH203-treated group compared with the model group, while medial area showed a modest change (Figures 5F–H).

To assess VSMC proliferation and migration *in vivo*, immunohistochemical staining was performed. The expression of MMP2 and PCNA was markedly increased in the model group, indicating enhanced VSMC migration and proliferation. In contrast, JPH203 treatment significantly reduced the expression of these markers (Figures 5I,J). Importantly, tissue-level inhibition of SLC7A5 activity by JPH203 was assessed by quantifying leucine levels in the vascular media. Balloon injury increased leucine accumulation in vascular tissue, whereas JPH203 treatment significantly reduced leucine levels, suggesting effective suppression of SLC7A5 transporter activity in the vascular wall (Figure 5K).

Furthermore, Western blot analysis demonstrated that balloon injury significantly increased the phosphorylation levels of PI3K and Akt, accompanied by upregulation of SLC7A5 expression. Importantly, JPH203 treatment effectively reduced the phosphorylation of PI3K and Akt, without affecting total protein levels (Figure 5L).

Collectively, these results indicate that pharmacological inhibition of SLC7A5 alleviates vascular remodeling and improves vascular function, at least in part through suppression of the PI3K/Akt signaling pathway *in vivo*. These findings are consistent with our *in vitro* results, further supporting a critical role of SLC7A5 in regulating vascular remodeling.

## 4 Discussion

### 4.1 SLC7A5: a novel metabolic driver underpinning pathological vascular remodeling

Pathological vascular remodeling is the unifying substrate of atherosclerosis and post-PCI in-stent restenosis (ISR), orchestrated by aberrant crosstalk between signaling cascades and metabolic reprogramming in VSMCs and the vascular microenvironment (Grobs et al., 2024; Wu et al., 2024; Wang et al., 2025). Herein, we establish definitive evidence that SLC7A5—a pivotal leucine/large neutral amino acid transporter—functions as a critical pro-pathological modulator of post-balloon injury remodeling, with its efficacy strictly dependent on PI3K/Akt pathway activation. These findings forge a direct causal link between amino acid transport and pro-proliferative signaling in vasculature, validate the metabolism-signaling coupling hypothesis in vascular pathology, and expand SLC7A5’s role beyond canonical nutrient transport.

SLC7A5 upregulation in injured vessels is a programmed adaptive response, not a stochastic event (Lili et al., 2020). Vascular mechanical injury elicits hypoxia, pro-inflammatory cytokines (TNF-α, IL-6), and mitogenic growth factors (PDGF-BB) (Zhao et al., 2021; Dutzmann et al., 2025), which converge to drive SLC7A5 transcription: HIF-1α binds its promoter HREs (Kang et al., 2024). This initial upregulation meets activated vascular cells’ leucine demand for early repair, but excessive SLC7A5-mediated leucine influx hyperactivates PI3K/Akt, triggering uncontrolled VSMC proliferation, migration, and ECM deposition that perpetuates remodeling. This dual role underscores metabolic adaptation’s double-edged sword in vascular injury, establishing SLC7A5 as a rational therapeutic target-with small-molecule inhibitors like JPH203 poised to interrupt this pro-remodeling axis.

### 4.2 Mechanistic basis of SLC7A5-mediated PI3K/Akt activation

In the present study, SLC7A5 is identified as a critical regulator of VSMC activation during vascular remodeling, with its effects closely linked to PI3K/Akt pathway activation. Both bioinformatic analyses and experimental validation demonstrate robust activation of PI3K/Akt signaling in vascular injury, which is significantly attenuated by genetic silencing or pharmacological inhibition of SLC7A5.

Mechanistically, SLC7A5 inhibition reduces PI3K and Akt phosphorylation without altering total protein expression, indicating that SLC7A5 regulates PI3K/Akt signaling primarily at the level of pathway activation. Consistent with the established role of PI3K/Akt signaling in VSMC proliferation, migration, and phenotypic switching, suppression of this pathway is accompanied by reduced VSMC activation.

Although the upstream mechanism remains incompletely defined, SLC7A5-mediated leucine transport may influence receptor tyrosine kinase signaling or nutrient-sensing pathways that converge on PI3K activation. Alternatively, metabolic reprogramming downstream of amino acid transport may contribute to modulation of VSMC phenotype (Arafath et al., 2021; Fedoroff et al., 2025). Further studies are warranted to define the precise molecular linkage between SLC7A5-dependent amino acid transport and PI3K/Akt signaling in vascular remodeling.

### 4.3 Translational potential of JPH203 for vascular remodeling-driven CVDs

The identification of SLC7A5 as a key regulator of vascular remodeling highlights its potential as a therapeutic target for cardiovascular diseases characterized by pathological vascular remodeling, including restenosis, atherosclerosis, and related vascular disorders (Jieyu et al., 2023; Xian et al., 2025). In the present study, pharmacological inhibition of SLC7A5 with JPH203 effectively suppressed VSMC proliferation, migration, and phenotypic switching *in vitro*, while significantly attenuating neointimal hyperplasia *in vivo*. These findings support a critical role for SLC7A5-mediated metabolic signaling in the regulation of pathological vascular responses following injury.

Importantly, the beneficial effects of JPH203 extended beyond structural alterations of the vessel wall. Treatment with JPH203 was also associated with improvements in vascular function, as reflected by enhanced hemodynamic parameters and reduced pathological remodeling burden (Naohiro et al., 2020; Yoshikatsu, 2021). These observations suggest that SLC7A5 inhibition may provide integrated therapeutic benefits by simultaneously preserving vascular architecture and improving functional outcomes.

From a translational perspective, JPH203 is particularly notable because it has already entered early-phase clinical evaluation in oncology, providing preliminary information on its pharmacokinetic profile and systemic exposure (Jieyu et al., 2023). However, dose-limiting toxicities observed at higher exposure levels indicate potential safety constraints, likely reflecting the fundamental role of SLC7A5 in transporting large neutral amino acids across multiple tissues. Given its broad expression and essential metabolic functions, systemic inhibition of SLC7A5 may disrupt amino acid homeostasis, thereby narrowing its therapeutic window for cardiovascular applications.

Therefore, the major challenge in translating SLC7A5 inhibition into cardiovascular therapy may not lie in target validation, but rather in achieving sufficient tissue specificity. Future strategies may focus on spatially or temporally controlled modulation of SLC7A5 activity, including vascular-targeted delivery systems, local administration approaches, or context-dependent inhibition strategies. Such approaches may maximize vascular protective effects while minimizing systemic metabolic disturbances.

Collectively, our findings position SLC7A5 as a promising upstream regulator and therapeutic target in vascular remodeling–associated cardiovascular diseases. However, successful clinical translation will likely require optimization of delivery strategies and treatment regimens to balance therapeutic efficacy with long-term metabolic safety.

From a translational perspective, JPH203 is notable for having already entered early-phase clinical evaluation in oncology, providing initial information on its pharmacokinetic behavior and systemic exposure. However, dose-limiting toxicities observed at higher exposure levels suggest potential safety constraints, likely attributable to the fundamental role of SLC7A5 in transporting large neutral amino acids across multiple tissues. Given its broad expression and essential metabolic functions, systemic inhibition of SLC7A5 may disrupt amino acid homeostasis, thereby narrowing its therapeutic window for cardiovascular applications.

Therefore, the major challenge for translating SLC7A5 inhibition into clinical cardiovascular therapy may not lie in target validation itself, but rather in achieving sufficient therapeutic specificity. Future strategies may benefit from approaches that enable spatially or temporally restricted modulation of SLC7A5 activity, such as vascular-targeted drug delivery systems, local administration strategies, or context-dependent inhibition paradigms. Such approaches may maximize vascular protective effects while minimizing systemic metabolic disturbances.

Collectively, our findings position SLC7A5 as a promising upstream regulator and therapeutic target in vascular remodeling–associated cardiovascular diseases. However, successful clinical translation will likely require optimization of delivery strategies and treatment regimens to balance therapeutic efficacy with long-term metabolic safety.

### 4.4 Limitations and future directions

Several limitations of the present study identify actionable directions for future research to refine our understanding of SLC7A5 and advance translational potential. First, our focus on acute rat carotid artery balloon injury models necessitates validation in chronic CVD models to recapitulate human disease’s long-term inflammation, lipid accumulation, and progressive remodeling. Second, while we confirm SLC7A5 drives remodeling via PI3K/Akt, the specific downstream effectors (e.g., cyclin D1, MMP9) targeted by SLC7A5 inhibition remain undefined, warranting multi-omics analyses to map the complete molecular footprint. Third, cell type-specific functional dissection—using VSMC-, endothelial cell-, and macrophage-specific SLC7A5 knockout mice—is needed to clarify which vascular cell populations mediate SLC7A5’s pro-remodeling effects.

To address these gaps, subsequent studies will adopt a translation-focused approach: (1) Integrate single-cell metabolomics/spatial transcriptomics to map SLC7A5-dependent metabolic reprogramming in human atherosclerotic/restenotic tissues, validating exogenous leucine’s role in reversing SLC7A5 knockdown-mediated PI3K/Akt suppression. (2) Define SLC7A5-PI3K/Akt crosstalk with metabolic stress pathways and leucine’s regulatory role to optimize JPH203’s efficacy in comorbid settings. (3) Evaluate JPH203 in preclinical in-stent restenosis models (e.g., pig drug-eluting stent studies) to validate its post-PCI utility for ISR prevention.

In summary, our study identifies SLC7A5 as a critical pro-remodeling regulator, elucidates the SLC7A5→PI3K/Akt→VSMC proliferation axis, and validates JPH203 as a potent inhibitor of this pathway. These findings advance understanding of metabolism-signaling coupling in vascular pathophysiology while providing a translational roadmap for JPH203’s repurposing to address post-PCI ISR and atherosclerotic progression. Despite the need for further preclinical/clinical validation, our work establishes a robust foundation for SLC7A5-targeted vascular therapies and opens new avenues for amino acid transporter inhibitors in CVD treatment.

## Data Availability

The raw data supporting the conclusions of this article will be made available by the authors, without undue reservation.

## Supplementary material

SUPPLEMENTARY FIGURE 1 Validation of SLC7A5 knockdown efficiency by siRNA. **(A)** Western blot analysis was performed to detect the protein expression of SLC7A5 in cells transfected with negative control siRNA (NC) or SLC7A5-targeted siRNA (siSLC7A5). Tubulin was used as the internal loading control. The bar graph shows the quantitative analysis of SLC7A5 protein levels normalized to Tubulin. **(B)** qRT-PCR was used to measure the relative mRNA expression of SLC7A5 in NC and siSLC7A5 groups. The data are presented as fold change relative to the NC group.All data are shown as the mean ± standard deviation (SD). Statistical significance was determined by unpaired Student’s t-test.

SUPPLEMENTARY FIGURE 2 Cytotoxic effect of JPH203 on vascular smooth muscle cells (VSMCs) determined by CCK-8 assay. **(A)** Cell viability of VSMCs treated with increasing concentrations of JPH203 (0, 0.1, 1, 10, 50, 100 μM) for the indicated time, measured by CCK-8 assay. Data are presented as the percentage of viable cells relative to the untreated control (0 μM) group. **(B)** Dose-response curve of JPH203-induced enzyme inhibition in VSMCs, plotted as enzyme inhibition percentage against log (concentration). The half-maximal inhibitory concentration (IC_50_) of JPH203 was calculated to be 63.36 μM via non-linear regression fitting. All data are shown as the mean ± standard deviation (SD) from at least three independent experiments.

SUPPLEMENTARY FIGURE 3 JPH203 inhibits the function of SLC7A5 in VSMCs. Relative leucine levels in the VSMCs were quantified and normalized to the Control group. PDGF-BB stimulation significantly increased leucine accumulation compared with the Control group, whereas treatment with JPH203 markedly attenuated the PDGF-BB–induced increase in leucine levels. Data are presented as mean ± SEM. Statistical significance was determined using one-way ANOVA followed by post hoc multiple comparisons. P values are indicated in the figure.

SUPPLEMENTARY FIGURE 4 TNF-α did not significantly alter the expression of SLC7A5 in ECs. Relative SLC7A5 levels in the ECs were quantified and normalized to the Control group. TNF-α stimulation did not influence SLC7A5 expression compared with the Control group (n=4). Data are presented as mean ± SEM. Comparisons between two groups were performed using an unpaired two-tailed Student’s t-test. ns indicates no significant difference.

## References

[B1] Arafath N. A. Fatih C. K. F. S. Gregory H. T S. R. Holly H. (2021). The amino acid transporter SLC7A5 is required for efficient growth of KRAS-Mutant colorectal cancer. J. Nat. Genet. 53 (1), 16–26. 10.1038/s41588-020-00753-3 33414552

[B2] Bolin W. Jinli P. Shengnan X. Jie L. Jinming Y. (2024). A glutamine tug-of-war between cancer and immune cells: recent advances in unraveling the ongoing battle. J. Exp. and Clin. Cancer Res. 43 (1), 74. 10.1186/s13046-024-02994-0 38459595 PMC10921613

[B3] Chen X. Zhong X. Luo D. Huang Q. Tang P. Ye L. (2026). Engeletin alleviates doxorubicin-induced cardiotoxicity via the AMPK pathway in mice. J. Front. Pharmacol. 17, 1741741. 10.3389/fphar.2026.1741741 PMC12979110 41836027

[B4] Dang W. Bian R. Fan Q. Cai C. Yun P. H. Song X. (2021). KIF11 promotes cell proliferation via ERBB2/PI3K/AKT signaling pathway in gallbladder cancer. J. Int. J. Biol. Sci. 17 (2), 514–526. 10.7150/ijbs.54074 33613109 PMC7893577

[B5] Dirk M. Sujin P. Michael N. H. (2018). mTOR Signalling and Cellular Metabolism Are Mutual Determinants in Cancer. Nat. Rev. Cancer 18, 744–757. 30425336 10.1038/s41568-018-0074-8

[B6] Du J. Yuan X. Wang J. Zhang L. Tan F. Hu T. (2025). The RNA-Binding protein RBPMS inhibits smooth muscle cell-driven vascular remodeling in atherosclerosis and vascular injury. J. Proc. Natl. Acad. Sci. U. S. A. 122 (9), e2415933122. 10.1073/pnas.2415933122 PMC11892686 39999164

[B7] Dutzmann J. Daniel J. M. Korte L. Kloss F. J. Knöpp K. Kalies K. (2025). Adventitial fibroblasts release interleukin 6 after vascular injury and induce smooth muscle cell proliferation and neointima formation. J. Am. Heart Assoc. 14, e040143. 10.1161/jaha.124.040143 40611486 PMC12533623

[B8] Estar S. M. Cidad P. Martinez M. A. Portillo A. M. Moraleda M. S. Alonso E. (2025). Vascular smooth muscle cell migration and P70S6K: key players in intimal hyperplasia development. J. Am. Heart Assoc. 14 (9), e038358. 10.1161/jaha.124.038358 40314369 PMC12184256

[B9] Fedoroff M. Y. Zhao L. Wang S. Bhushan A. Yang H. Bussard K. M. (2025). Amino acid transporter LAT1 (SLC7A5) promotes metabolic rewiring in TNBC progression through the L-Trp/QPRT/NAD+ pathway. J. Exp. Clinical Cancer Res. CR. 44 (1), 190. 10.1186/s13046-025-03446-z 40611146 PMC12224598

[B10] Gao C. Huang Q. Liu C. Kwong C. H. T. Wang R. J. N. C. Wan J. B. (2020). Treatment of atherosclerosis by macrophage-biomimetic nanoparticles via targeted pharmacotherapy and sequestration of proinflammatory cytokines. Nat. Commun. 11 (1), 2622. 10.1038/s41467-020-16439-7 32457361 PMC7251120

[B11] Grobs Y. Romanet C. Lemay S. E. Bourgeois A. Voisine P. Theberge C. (2024). ATP citrate lyase drives vascular remodeling in systemic and pulmonary vascular diseases through metabolic and epigenetic changes. Sci. Translational Med. 16 (777), eado7824. 10.1126/scitranslmed.Ado7824 39661707

[B12] Jianqiu P. Lin C. Fang W. Chuansheng X. Shengqiang P. Hongwei G. (2022). LPA2 contributes to vascular endothelium homeostasis and cardiac remodeling after myocardial infarction. Circulation Res, 131, 5. 10.1161/circresaha.122.321036 35920162

[B13] Jieyu G. Jingjing Q. Mengping J. Qinhan L. Xiangxiang W. Liliang L. (2023). BACH1 deficiency prevents neointima formation and maintains the differentiated phenotype of vascular smooth muscle cells by regulating chromatin accessibility. Nucleic Acids Res. 51 (9). 10.1093/nar/gkad120 PMC10201429 36864760

[B14] Kang Y. J. Song W. Lee S. J. Choi S. A. Chae S. Yoon B. R. (2024). Inhibition of BCAT1-mediated cytosolic leucine metabolism regulates Th17 responses via the mTORC1-HIF1α pathway. Exp. and Mol. Med. 56 (8), 1776–1790. 10.1038/s12276-024-01286-z 39085353 PMC11372109

[B15] Lahmann A. L. Joner M. (2020). In-Stent restenosis. JACC Basic Transl. Sci. 5 (1), 12–14. 10.1016/j.jacbts.2019.12.002 32043490 PMC7000883

[B16] Lili Q. Ryuichi O. Saori H. Suguru O. Ling W. Hiroki O. (2020). Amino acid transporter LAT1 in tumor-associated vascular endothelium promotes angiogenesis by regulating cell proliferation and VEGF-A-dependent mTORC1 activation. J. Exp. and Clin. Cancer Res. 39 (1), 266. 10.1186/s13046-020-01762-0 33256804 PMC7702703

[B17] Marissa D. P. David P. M. Panfeng F. Clara F. M. Hoshang U. Kim T. (2023). Metabolic reprogramming, oxidative stress, and pulmonary hypertension. J. Redox Biol. 64, 102797. 10.1016/j.Redox.2023.102797 PMC10363484 37392518

[B18] Martina W. Jörg K. Julia H. Albrecht E. Cordula A. A K. H. (2005). Metabolic control analysis of the Warburg-effect in proliferating vascular smooth muscle cells. J. Biomed. Sci. 12 (5), 827–834. 10.1007/s11373-005-9010-5 16205843

[B19] Matsushita K. Sato C. Bruckert C. Gong D. Amissi S. Hmadeh S. (2024). Potential of dapagliflozin to prevent vascular remodeling in the rat carotid artery following balloon injury. J. Atheroscler. 397, 117595. 10.1016/j.Atherosclerosis.2024.117595 38879387

[B20] Nathon D. W. Naveed S. (2023). Cardiovascular risk in diabetes mellitus: epidemiology, assessment and prevention. J. Nat. Reviews. Cardiol. 20 (10), 685–695. 10.1038/s41569-023-00877-z 37193856

[B21] Naohiro O. Daisuke N. Kirio K. Takaaki K. Fumio N. Hitoshi E. (2020). First-in-human phase I study of JPH203, an L-type amino acid transporter 1 inhibitor, in patients with advanced solid tumors. J. Investig. New Drugs 38 (5), 1–12. 10.1007/s10637-020-00924-3 32198649

[B22] Nicole Z. L F. A. Zhiying X. P B. L. K W. R. E M. C. (2023). Stacking thick perfusable human microvascular grafts enables dense vascularity and rapid integration into infarcted rat hearts. J. Biomater. 301, 122250. 10.1016/j.Biomaterials.2023.122250 PMC10530304 37481833

[B23] Qi D. Wei M. Jiao S. Song Y. Wang X. Xie G. (2019). Hypoxia inducible factor 1α in vascular smooth muscle cells promotes angiotensin II-induced vascular remodeling via activation of CCL7-mediated macrophage recruitment. Cell Death Dis. 10 (8), 1–15. 10.1038/s41419-019-1757-0 31320613 PMC6639417

[B24] Qingwei W. Gulcin O. H. Bowen W. Mengxue Z. Go U. Yitao H. (2021). A hierarchical and collaborative BRD4/CEBPD partnership governs vascular smooth muscle cell inflammation. Mol. Ther. Methods and Clin. Dev. 21 (prepublish), 54–66. 10.1016/j.Omtm.2021.02.021 33768129 PMC7966960

[B25] RuLi L. CaiLi Z. Xin Y. He L. Lan L. LingYu L. (2024). Irisin attenuates vascular remodeling in hypertensive mice induced by ang II by suppressing Ca2+-dependent endoplasmic reticulum stress in VSMCs. Int. J. Biol. Sci. 20 (2), 680–700. 10.7150/ijbs.84153 38169582 PMC10758105

[B26] Tong Z. Qingxun H. Yanggang Y. Huijuan Y. Jian Z. Jia Q. (2023). Mitochondrial dynamics in vascular remodeling and target-organ damage. Front. Cardiovasc. Med. 10, 1067732. 10.3389/fcvm.2023.1067732 36860274 PMC9970102

[B27] Wang Y. Zhang X. Li X. Cheng M. Cui X. (2025). The vascular microenvironment and its stem cells regulate vascular homeostasis. Front. Cell Dev. Biol. 13, 1544129. 10.3389/fcell.2025.1544129 40114970 PMC11922910

[B28] Wenjuan M. Yanling W. Rongxin Z. Fan Y. Duo Z. Menggui H. (2020). Targeting PAK4 to reprogram the vascular microenvironment and improve CAR-T immunotherapy for glioblastoma. J. Nat. Cancer 2 (1), 83–97. 10.1038/s43018-020-00147-8 PMC10097424 35121889

[B29] Wu Y. Chen Z. Zheng Z. Li X. Shu J. Mao R. (2024). Tudor-SN exacerbates pathological vascular remodeling by promoting the polyubiquitination of PTEN via NEDD4-1. J. Biomed. Sci. 31 (1), 88. 10.1186/s12929-024-01076-9 39237902 PMC11378411

[B30] Xian G. Pan H. Sha Z. Qiang X. Mingyao Z. Wendong T. (2025). Enhancer-associated LncRNA-ITGA2 promotes vascular remodeling through ITGA2. Circulation Res. 136 (12), 1610–1628. 10.1161/circresaha.124.325443 40321134

[B31] Xinyue H. Jiayan G. Anqi N. Naijin Z. Yingxian S. (2024). BAG3 promotes proliferation and migration of arterial smooth muscle cells by regulating STAT3 phosphorylation in diabetic vascular remodeling. J. Cardiovasc. Diabetol. 23 (1), 140. 10.1186/s12933-024-02216-z PMC11046803 38664681

[B32] Yang J. Ma Z. Wan W. Yuan D. Li J. Wang Y. (2026). The hippo-YAP1/TEAD1-SLC7A5 axis: uncovering a novel therapeutic target for oxalate-induced renal tubular ferroptosis. Redox Rep. Commun. Free Radic. Research 31 (1), 2643967. 10.1080/13510002.2026.2643967 41833934 PMC12990274

[B33] Yoshikatsu K. (2021). Amino acid transporter LAT1 (SLC7A5) as a molecular target for cancer diagnosis and therapeutics. Pharmacol. and Therapeutics 230, 107964. 10.1016/j.Pharmthera.2021.107964 34390745

[B34] Yu C. Chen Y. Luo H. Lin W. Lin X. Jiang Q. (2024). NAT10 promotes vascular remodelling via mRNA ac4C acetylation. Eur. Heart Journal 46, 288–304. 10.1093/eurheartj/ehae707 39453784

[B35] Zenglei Z. Lin Z. Xingyu Z. Xu M. Xianliang Z. (2023). Role of inflammation, immunity, and oxidative stress in hypertension: new insights and potential therapeutic targets. Front. Immunol. 13, 1098725. 10.3389/fimmu.2022.1098725 36703963 PMC9871625

[B36] Zhang J. Wang X. Fu Z. Xing C. Wang Z. Yang H. (2024). Long-term simulated microgravity fosters carotid aging-like changes via Piezo1. Cardiovasc. Res. 120, 548–559. 10.1093/cvr/cvae024 38271270

[B37] Zhang M. Shi J. Pan H. Zhu J. Wang X. Zhou J. (2024). F-53B stimulated vascular smooth muscle cell phenotypic switch and vascular remodeling via ferroptosis-related pathway. Sci. Total Environ. 954, 176565. 10.1016/j.Scitotenv.2024.176565 39341237

[B38] Zhang Y. Kong X. Liang L. Xu D. (2024). Regulation of vascular remodeling by immune microenvironment after the establishment of autologous arteriovenous fistula in ESRD patients. Front. Immunol. 15, 1365422. 10.3389/fimmu.2024.1365422 38807593 PMC11130379

[B39] Zhao M. Wang S. Zuo A. Zhang J. Wen W. Jiang W. (2021). HIF-1α/JMJD1A signaling regulates inflammation and oxidative stress following hyperglycemia and hypoxia-induced vascular cell injury. Cell Mol. Biol. Letters 26 (1), 40. 10.1186/s11658-021-00283-8 34479471 PMC8414688

[B40] Zheng H. Zhang X. Li C. Wang D. Shen Y. Lu J. (2024). BCAA mediated microbiota-liver-heart crosstalk regulates diabetic cardiomyopathy via FGF21. J. Microbiome 12 (1), 157. 10.1186/s40168-024-01872-3 39182099 PMC11344321

[B41] Zhou M. Wang H. Zeng X. Yin P. Zhu J. Chen W. (2020). Mortality, morbidity, and risk factors in China and its provinces, 1990-2017: a systematic analysis for the global burden of disease study 2017 (vol 394, pg 1145, 2019). J. Lancet 396 (10243), 26. 10.1016/S0140-6736(19)30427-1 PMC6891889 31248666

[B42] Zhou Z. Zhang B. Deng Y. Deng S. Li J. Wei W. (2024). FBW7/GSK3β mediated degradation of IGF2BP2 inhibits IGF2BP2-SLC7A5 positive feedback loop and radioresistance in lung cancer. J. Exp. Clinical Cancer Res. CR 43 (1), 34. 10.1186/s13046-024-02959-3 38281999 PMC10823633

