# Supplementary figures and images for: SLC7A5 promotes vascular remodeling in the rat carotid artery following balloon injury through PI3K/Akt signaling pathway
Source: Front Pharmacol. 2026 Jul 14;17:1857850. doi: 10.3389/fphar.2026.1857850 (PMC13407181; doi:10.3389/fphar.2026.1857850)

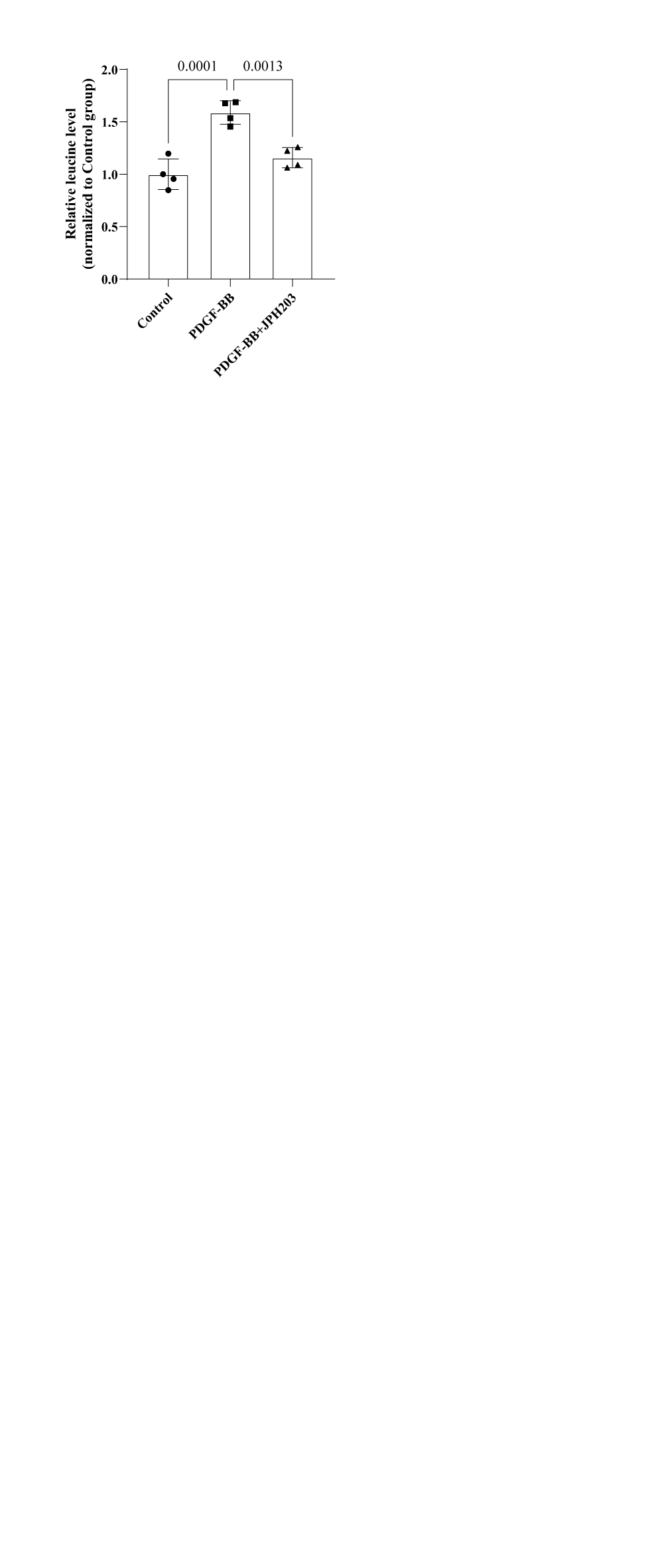

Supplement: Supplementary file 1 [file Image3.jpeg]

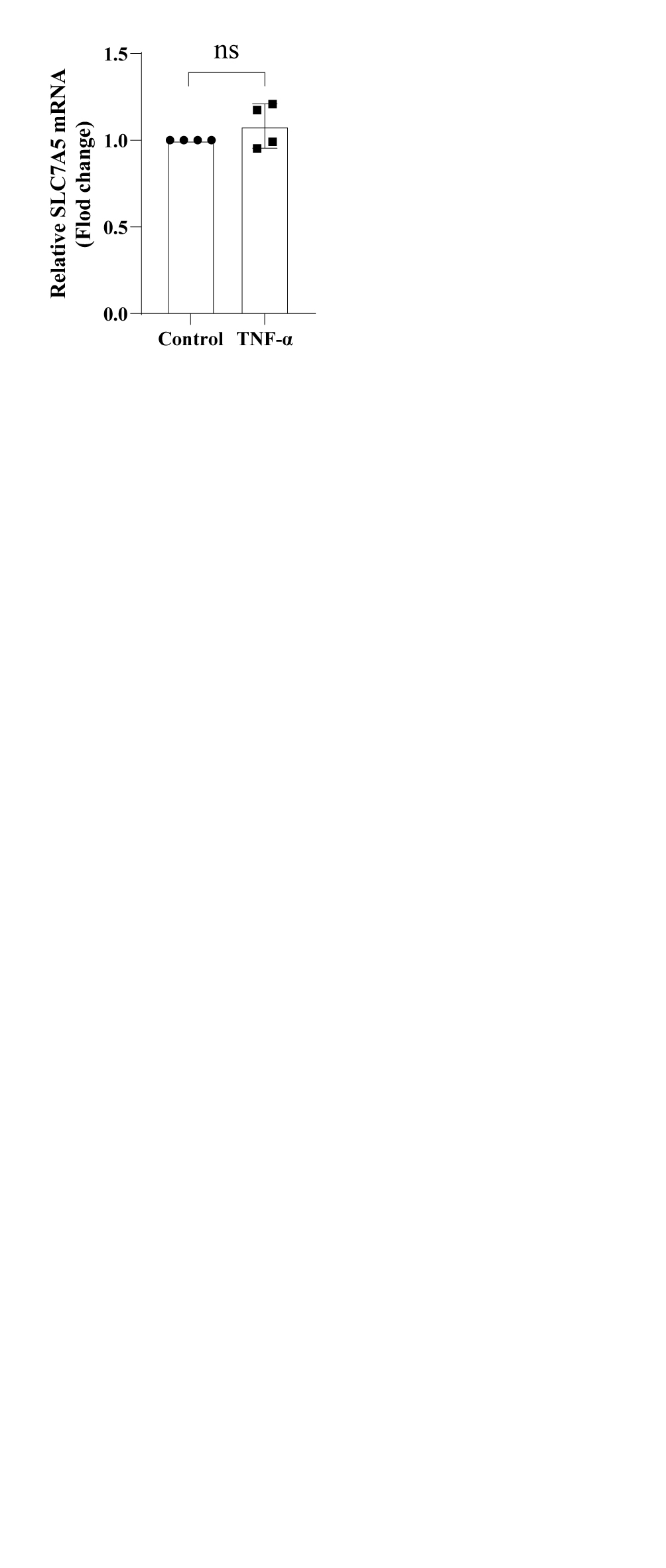

Supplement: Supplementary file 2 [file Image4.jpeg]

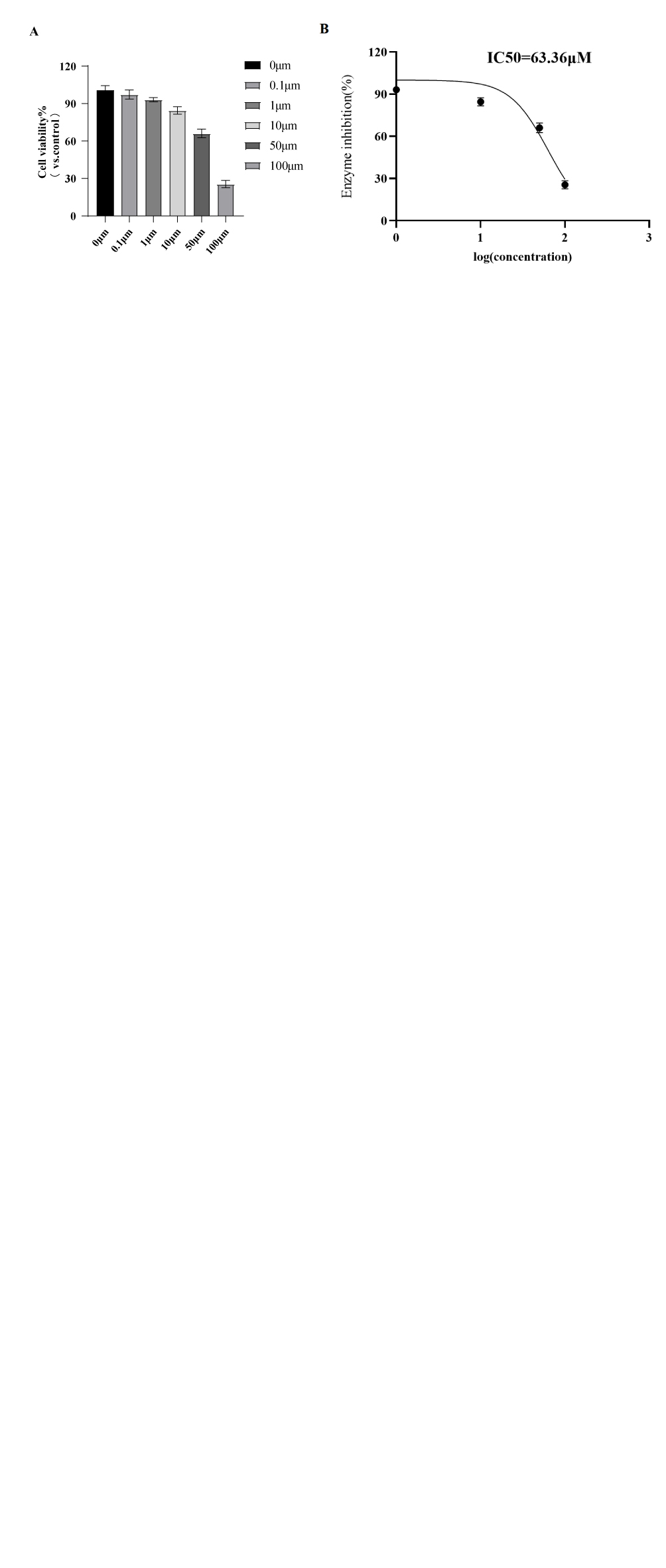

Supplement: Supplementary file 3 [file Image2.jpeg]

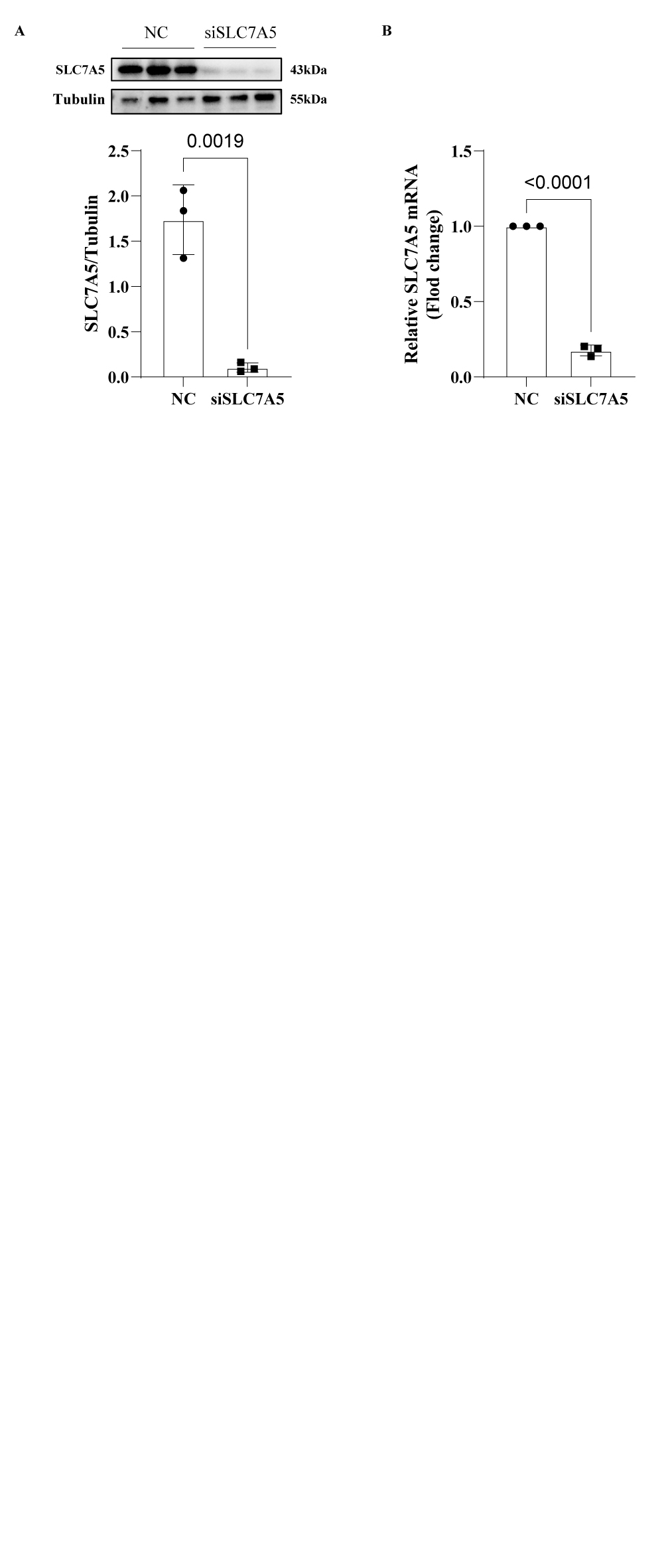

Supplement: Supplementary file 4 [file Image5.jpeg]

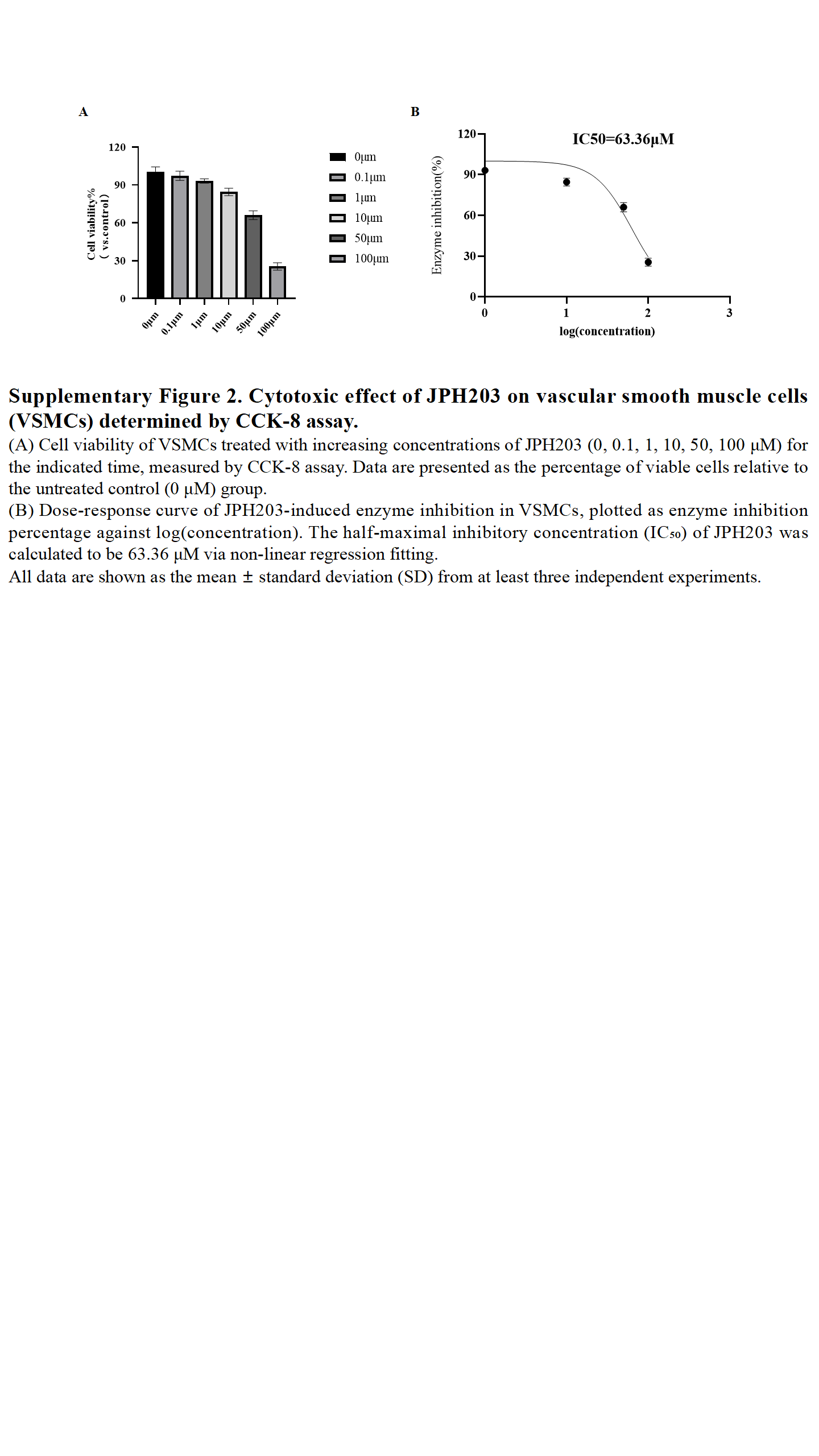

Supplement: Supplementary file 5 [file Image1.tif]
